# From Aspiration to Admission: Understanding Pre‐Medical Student Perceptions and Decision‐Making Processes

**DOI:** 10.1111/tct.70478

**Published:** 2026-07-18

**Authors:** L. Canthiya, M. Wang, A. Bouriayee, R. Qamar, A. Acai, Preti BTB

**Affiliations:** ^1^ Schulich School of Medicine & Dentistry Western University London Ontario Canada; ^2^ Princess Margaret Cancer Center Toronto Ontario Canada; ^3^ University of Ottawa Faculty of Medicine Ottawa Ontario Canada; ^4^ Department of Psychiatry and Behavioural Neurosciences McMaster University, McMaster Education Research, Innovation and Theory (MERIT) Centre Hamilton Ontario Canada; ^5^ Department of Oncology Western University London Ontario Canada; ^6^ Department of Haematology & Medical Oncology Emory University Atlanta Georgia USA; ^7^ School of Health Professions Education Maastricht University Maastricht the Netherlands

**Keywords:** pregnancy discrimination spillover–crossover, social‐cognitive career theory, work self‐efficacy

## Abstract

**Background:**

The medical school application process is demanding, with several factors contributing to burnout. Existing literature highlights pre‐medical students' perceptions about what is required for admissions success. These perceptions can drive extreme behaviours; however, program‐level evidence shows such steps may not always be necessary or desired. This discrepancy underscores the need to explore the origins of these perceptions, improve guidance, reduce burnout, and promote a balanced pre‐medical experience.

**Methods:**

This generic qualitative study, informed by a constructive orientation, used reflexive thematic analysis. Twelve pre‐medical students were recruited through online forum posts and snowball sampling. Semi‐structured interviews explored how participants constructed perceptions of admissions success and how this informed decision making. Analysis involved regular reflexive team discussions.

**Findings:**

Four themes were developed: a) Formal and informal influences shape pre‐medical students' perceptions and interpretations of the application process; b) Early, performative strategizing is used to navigate the perceived competitive system; c) Trial, adaptation and reflection occur iteratively to reflect an ideal medical student ‘archetype,’ and d) The medical school application process elicits tensions between authenticity and ‘what the system wants.’

**Conclusion:**

Findings suggest that pre‐medical students construct perceptions of admissions success through an iterative process of information gathering, reflection and adaptation. These perceptions contribute to the development of an ‘ideal’ applicant archetype that shapes strategic self‐presentation and tensions between authenticity and perceived admissions expectations. Our findings highlight a need for more transparent outreach opportunities between the medical community and pre‐medical students to avoid echo chambers and reduce inauthenticity and burnout.

## 1 Background

The academic and professional journey to becoming a physician begins well before matriculation; the first major hurdle is applying to medical school. Admission has become increasingly competitive in North America, with acceptance rates steadily declining over the past decades. In Canada, the matriculation rate in the early 1970s was approximately 50%, which dropped to 20% in the 1998–1999 application cycle [1] and 7.6% in the 2024–2025 cycle [2]. Given this highly competitive context, the application process can be arduous for students and contribute to burnout.

Pre‐medical students often perceive medical schools as prioritizing high grades, strong Medical College Admission Test (MCAT) scores and extensive extracurricular involvement [3]. These perceptions can contribute to academic and psychological stress, overloaded schedules and emotional exhaustion [4].

Despite admission to medical school being highly competitive, there is no consensus in the literature on the impact of factors such as grade point average (GPA), MCAT score and extra‐curricular involvement on medical school admission success, in part because medical schools do not uniformly prioritize these prerequisites. While some studies found that higher GPA and MCAT score increase the likelihood of acceptance, this relationship varies across race/ethnicity, socioeconomic status and geographic region of the applicant [5]. Furthermore, program‐level evidence from the program perspective indicates that admissions committees increasingly value the context and quality of applicants' experiences over academic overload [6].

Existing literature has explored the influence of formal and informal factors influencing medical school admissions, including professional identity formation [7, 8]. Concepts such as anticipatory socialization and the hidden curriculum describe how people adopt professional values, behaviours and expectations of success before beginning their career [9, 10]. However, few studies have integrated these concepts to examine how pre‐medical students form professional identities in anticipation of admission. Furthermore, it is unclear how students develop perceptions of which strategies contribute to admissions success or why students pursue specific strategies despite potential discrepancies with admissions expectations.

To address this gap, our study aimed to explore pre‐medical students' perceptions of success, including how such views are constructed and internalized and how these views inform decision making for the application process.

## 2 Methods

A generic qualitative research approach was selected to explore participants' individual thoughts and experiences [11]. Informed by a constructivist orientation, understandings of admissions success were regarded as shaped by participants' experiences, interpretations and interactions with formal and informal sources of information [12, 13]. This allowed us to examine how perceptions influenced decision making without assuming they directly reflect formal admissions criteria. Reflexive thematic analysis was undertaken following Braun and Clarke's six‐phase approach, treating researcher subjectivity as a resource for developing interpretations rather than discovering pre‐existing themes [11, 14]. Team reflection considered how positions and experiences within medical education shaped interpretation.

The research team consisted of medical students, undergraduate students, an academic medical oncologist and medical education researcher and a PhD‐trained health professions education researcher and applied health research methodologist. Interviews were conducted by members who were closest to participants' stage of training. Pilot interviews were practised between these authors prior to data collection, overseen by the principal investigators.

### 2.1 Participant recruitment and data collection

Participants were recruited through posts on the Premed Canada subreddit, a Reddit forum frequently visited by pre‐medical students, which included a study description and a sign‐up link. Snowball sampling was used to expand respondent reach.

### 2.2 Data collection and analysis

Semi‐structured interviews were conducted virtually via Microsoft Teams and ranged from 45–60 min in duration. Interviews were audio‐recorded and automatically transcribed using Teams before being manually reviewed for accuracy. Interviews followed a funnelling approach, beginning with broad questions and progressing to more specific probes (as cited in [15]). The interview guide is provided in Appendix 1.

Data collection and analysis occurred iteratively. After analysis of nine interviews, the team reviewed the developing analysis and conducted three further interviews to finalize the dataset, resulting in a final sample of 12 participants.

Consistent with Braun and Clarke's approach, one team member familiarized themselves with the transcripts before inductively coding all interviews and maintaining an evolving record of codes and code definitions. At intervals, coded excerpts and the developing analytic record were shared with all members of the research team. Team discussions considered code labels, alternative interpretations and connections across the dataset to refine ongoing reflexive analysis. For example, the team recognized that some participants shared understandings that appeared discrepant from formal admissions processes. We reflected on how our positions within medical educations influenced our interpretations of these statements. Consistent with the constructivist orientation, we examined these accounts not solely in terms of their factual accuracy, but as informal, constructed narratives of the admissions process that shaped participants' decision making.

One team member subsequently generated candidate themes by organizing related codes and the team discussed the conceptual boundaries, meanings and wording of these themes, informing their definition and naming. Reflexive discussion continued during manuscript writing to refine the analytic narrative and presentation of themes.

### 2.3 Ethics Statement

The study received approval from the institutional research ethics board (Project #125755). Participants were informed that interviews would be audio recorded and transcribed, that audio recordings would be deleted following transcription and identifying information would be removed from transcripts. De‐identified transcripts were stored in a password‐protected folder accessible only to one member of the research team. All participants provided verbal informed consent prior to participation.

## 3 Findings

Twelve interviews were conducted with pre‐medical students across Canada (Table 1).

**TABLE 1:** Participant demographic details.

Demographic detail	Description
Number of participants	12
Sex	Female: 7 Male: 5
Educational stage	Undergraduate level: 9 Graduate level: 1 Professional level: 2

**FIGURE 1:**
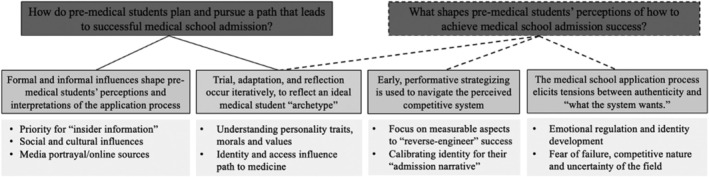
Conceptual model linking the research questions (top row) to the four themes (second row) and their descriptions (third row), derived from pre‐medical student interviews.

The four themes developed from the interview data are shown in Figure 1. These themes are: a) Formal and informal influences shape pre‐medical students' perceptions and interpretations of the application process; b) Early, performative strategizing is used to navigate the perceived competitive system; c) Trial, adaptation and reflection occur iteratively to reflect an ideal medical student ‘archetype,’ and d) The medical school application process elicits tensions between authenticity and ‘what the system wants.’ Each theme is described below.

### 3.1 Formal and Informal Influences Shape Pre‐Medical Students' Perceptions and Interpretations of the Application Process

Students described actively seeking external resources to understand the admissions process, citing a lack of transparency around expectations. One participant described this reliance as a way of navigating ‘unwritten rules’:

I feel like there's a lot of unwritten rules when it comes to applying to medicine … you don't really know what exactly is being looked for … being able to go online and see other people … talk about what they did or what program they did … that helps…. (P1)

Many students relied on conversations with medical professionals and family, community expectations and published admissions data, particularly insights from medical professionals, students and admissions data.

I look for something that's peer reviewed. A lot of the things that I'm also reading, like on news and whatnot, are opinion pieces. So, for sure, those have their own biases…but I try to read both sides of things…then based off that, I make my own conclusions based on what I've personally observed. (P2)

However, students also placed considerable weight on informal sources, including Reddit, social media, artificial intelligence (AI) tools and portrayals of medicine in popular media, with some identifying these as their primary sources of information.

I get a lot of my info from like Reddit and LinkedIn and whatever, and those Instagram med influencers. (P12)

Although students attempted to ‘vet’ these sources, interpretations were often based on conjecture and peer accounts, contributing to a range of perceptions and interpretations of admissions expectations. For example, some students decided research was an integral component of a competitive application based on observations of practicing physicians and anecdotal reports from previously successful applicants, despite limited interest in research.

*Although students attempted to ‘vet’ these sources, interpretations were often based on conjecture and peer accounts, contributing to a range of perceptions and interpretations of admissions expectations.*

I got into research because I know that that's another thing that [medical school admissions] like to see. [It's] not something that I went into because I altruistically wanted to do it … Anecdotally, people who did research say, hey, I got into medicine. (P10)

### 3.2 Early, Performative Strategizing Is Used to Navigate the Perceived Competitive System

Students described forming strategies years in advance, reflecting perceptions of the admissions process as highly competitive and uncertain. Many strategically analysed GPA, MCAT and Computer‐Based Assessment for Sampling Personal Characteristics (CASPer) scores to maximize perceived competitiveness when planning academic and extracurricular activities.

*Students described forming strategies years in advance, reflecting perceptions of the admissions process as highly competitive and uncertain*.

I would see admitted people with the [tag] “Admitted MD” [on Reddit] and then they would talk about their numerical stats, specific experiences and the interview … I internalized like, I need to have these things to match the chance of somebody who did get admitted. (P4)

Undergraduate program selection was often framed strategically, with students describing their choice of degree as a crucial decision aimed at optimizing perceived competitiveness for medical school admissions. Specifically, participants stated prioritizing programs that would allow them to maintain a high GPA while also providing time and flexibility for extracurriculars, rather than interest in the subject matter. This reflected the perception that success in medical school admissions depended not only on admissions metrics, but also on the strategic planning of time and activities throughout undergraduate training.

In Grade 12, I applied to the best possible programs … programs where I felt that I could achieve a high GPA and give myself more free time to like, study or do my extracurriculars … so I guess, I am lucky to be in a program that kind of inflates my GPA and gives me plenty of free time to do extracurriculars. (P6)

Students also pursued verifiable extracurricular activities to craft a strong admission ‘narrative,’ reflecting a performative approach to self‐presentation aligned with perceived admissions expectations.

I planned everything in university around my med application, like deciding whether it would or would not help my med application … I always try to do it in a way where it would be in some official capacity, and I could get it verified if that makes sense. (P6)

### 3.3 Trial, Adaptation and Reflection Occur Iteratively, to Reflect an Ideal Medical Student Archetype

Students evolved through a continuous process of trial, adaptation and reflection to identify strengths and growth areas.

I have always had an interest in science and the human body, just something that I've always really loved … that pushed me to be involved in science. (P7)

Some participants reflected on their personal experiences as patients influencing their understanding of the role of a physician. Through these clinical encounters, participants described how they developed an appreciation for the importance of trust and communication among physician‐patient interactions. These interpretations contributed to their growing understanding of the professional and interpersonal qualities that ‘should be’ associated with being a good physician.

I'm pretty sure we've all had doctors or healthcare workers who we didn't really feel comfortable sharing everything with … A physician is put in a place of high trust, and it's important that they know your entire history in order to create the best plan [for you, and not] hinder their ability to do their job. (P2)

While participants described diverse values and life experiences, many felt pressure to align with a perceived ideal medical student ‘archetype.’ They described becoming increasingly aware of the informal expectations surrounding the types of activities and achievements linked with previously successful applicants. Consequently, some selectively participated in experiences that would best align with these expectations, despite not completely reflecting their genuine interests. This process often came at the expense of self‐exploration and identity formation.

I've made conscious efforts to do more things only because I think that they will make me a good applicant and some volunteer work because I don't do a lot of volunteer work. (P10)

Students described refining their narratives by selectively choosing activities to stand out from peers, characterizing some commonly pursued experiences as ‘cookie‐cutter’ while simultaneously feeling compelled to engage in them.

A lot of the activities that people do where they are like crisis workers, or they do research for one summer are too cookie‐cutter … like [admissions committees] want you to have something that's unique. So, it's kind of like tokenifying my hobbies and passions. (P11)

### 3.4 The Medical School Application Process Elicits Tensions Between Authenticity and ‘What the System Wants’

Students described tensions between maintaining authenticity and aligning with perceived admissions expectations, impacting mental well‐being and self‐concept. Comparisons with peers amplified self‐doubt, while unwritten rules and timelines were described as unrealistic.

*Students described tensions between maintaining authenticity and aligning with perceived admissions expectations, impacting mental well‐being and self‐concept.*

I'll be seeing people posting [on Reddit] about, you know, I got a 528, and my GPA is perfect, and now I've been rejected from all these schools … why? And it gets kind of almost not even discouraging but then you get a little bit annoyed. (P5)

Some students felt pressured to disclose traumatic or deeply personal experiences in hopes of appearing genuine and unique, highlighting the emotional strain between remaining authentic and framing experiences to align with perceived admissions expectations.

I really hated writing essays. I feel like you really have to frame it in a way that makes your experiences seem a lot more like a lot of a bigger deal than [they] actually [are]. (P12)

## 4 Discussion

Our study explored pre‐medical students' perceptions of the medical school admissions process and how these perceptions influence decision making for achieving success in admissions. Rather than solely preparing to meet formal admissions requirements, participants' strategies reflected early identity formation evolving in an environment perceived to be opaque and highly competitive. Our constructivist analysis examined how participants made sense of admissions success within this environment, recognizing that their accounts, regardless of their alignment with formal admissions criteria, were consequential in shaping participants' academic planning, activity selection, self‐presentation and developing sense of an ‘ideal’ applicant.

*Rather than solely preparing to meet formal admissions requirements, participants' strategies reflected early identity formation evolving in an environment perceived to be opaque and highly competitive.*

**FIGURE 2:**
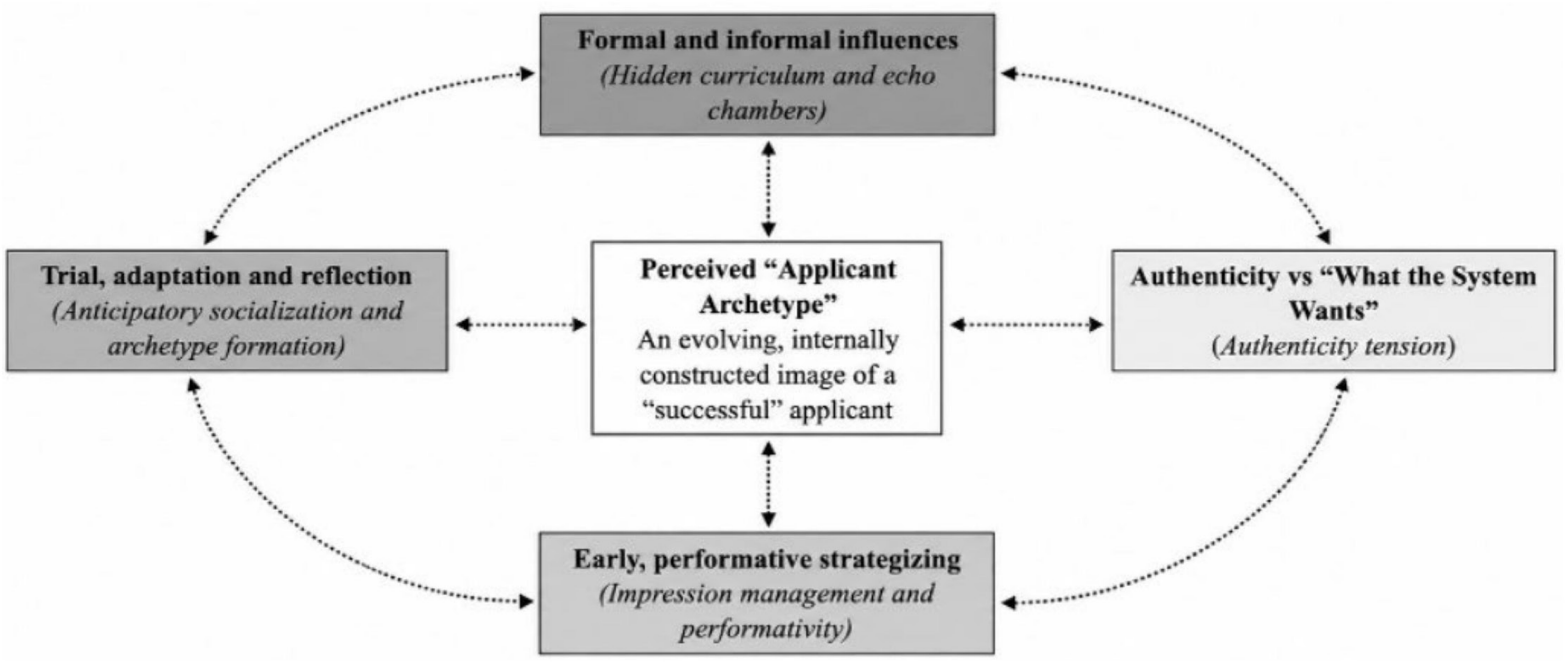
Interpretive conceptual framework synthesizing relationships among the four themes and corresponding theoretical constructs. The framework represents overlapping and potentially reciprocal relationships across participants' accounts, rather than a sequential model of participant decision making.

The four themes in Figure 1 highlight how participants interpreted beliefs about a ‘successful’ application, which may have informed their developing personal and professional identities. Rather than representing a linear or universal sequence of participant decision making, the framework in Figure 2 illustrates an interpretive synthesis of the relationships across the themes and relevant theoretical concepts, including the hidden curriculum, anticipatory socialization, impression management and performativity.

Although the hidden curriculum and its impact on medical trainees have been well described, our findings suggest that this concept may also be useful in understanding the pre‐medical space. In a United Kingdom study, medical students reported modifying their professional person as they became more aware of the ‘loose set of behaviours and attitudes’ associated with the hidden curriculum [16], similar to the archetype described in our study.

Our findings suggest that participants in this sample constructed overlapping perceptions of qualities associated with a competitive pre‐medical applicant through formal sources, such as admissions data and informal sources, such as social media forums. These qualities included academic excellence, extracurricular involvement, research engagement and interpersonal qualities, such as empathy and collaboration. Taken together, these accounts pointed to a perceived ‘applicant archetype’: an informal image of the qualities thought to characterize a competitive applicant, rather than a formally articulated model of admissions success.

*Taken together, these accounts pointed to a perceived ‘applicant archetype’: an informal image of the qualities thought to characterize a competitive applicant, rather than a formally articulated model of admissions success.*

Participants did not describe these qualities as consistently articulated in official admissions materials. Instead, some reported drawing on perceiving unwritten rules when considering their activities and self‐presentation. Participants actively synthesized formal admissions data with informal narratives obtained from external sources. This suggests that some participants perceived an implicit evaluative standard as influential in their decision making, although our data cannot establish the extent to which these perceptions reflected formal institutional expectations. Despite participants drawing on individual reflection and varied sources of information, participants' accounts included overlapping perceptions of qualities associated with an ideal applicant archetype.

This process may reflect anticipatory socialization, as participants used informal networks to interpret what they experienced as limited official guidance [10]. Participants described tension between remaining authentic and aligning with inferred admissions expectations while distinguishing themselves from other applicants. Some participants described adapting their self‐presentation or activities in response to these perceptions.

Moreover, participants described using informal sources, including social media and online forums, to fill perceived gaps in published admissions information. While these spaces may foster support and community, they may also amplify unverified advice through ‘echo chamber’ effects [17]. In this context, students may attempt to gain control over an uncertain process by following overgeneralized advice that appears to offer a tangible pathway to success, even when no guaranteed pathway exists. For example, one participant perceived research involvement as universally expected of clinicians, despite evidence that only a minority of physicians report research engagement [18]. This account may reflect performative pressures within the application process and align with prior literature suggesting that higher education may be pursued to meet perceived expectations rather than personal growth [19]. Such pressures may create tensions around authentic self‐presentation and contribute to stress and burnout.

*In this context, students may attempt to gain control over an uncertain process by following overgeneralized advice that appears to offer a tangible pathway to success, even when no guaranteed pathway exists.*

Our findings have implications for both pre‐medical students and medical schools. As perceptions of an ideal applicant archetype propagate, there is a risk of homogenizing the diversity of applicants and matriculants, in terms of belief systems and subsequent actions and activities [20]. First, addressing common misconceptions through myth‐versus‐fact resources, webinars and frequently asked questions may improve the transparency surrounding medical school admissions expectations. Second, admissions offices and outreach teams could provide targeted workshops and mentorship opportunities to showcase the diversity of experiences among successful applicants. Third, pre‐medical advisors and mentorship programs may help reduce performative pressures by incorporating structured self‐reflection tools and career exploration exercises that encourage students to align their activities with their genuine interests, values and long‐term career goals, rather than selecting experiences solely to enhance perceived competitiveness. Importantly, medical schools could continue to refine admissions processes that place less emphasis on GPA alone and more explicitly recognize authenticity, diverse life experiences and broader applicant contexts [21]. Together, these strategies may help foster a more authentic, informed and less stressful pre‐medical experience.

*As perceptions of an ideal applicant archetype propagate, there is a risk of homogenizing the diversity of applicants and matriculants, in terms of belief systems and subsequent actions and activities.*

Limitations of our study include potential selection bias and a single time‐point design. Participants were recruited through Reddit forums and snowball sampling. This recruitment approach captured the perspectives of students who use online platforms to inform their understanding of the medical school admissions process. However, this may have overrepresented people who were more actively engaged with online admissions discourse, which may have increased the prominence of the ‘echo chamber’ narratives identified in our findings. Voluntary participation may have overrepresented students interested in discussing medical school admissions. Additionally, since our data capture perceptions at one stage of the pre‐medical journey, they do not reflect how these perceptions may evolve over time. Moreover, we cannot determine how the perceived applicant archetype may influence professional identity formation during medical training. Future work could examine perceptions across institutions and longitudinally follow how these beliefs evolve as students' progress through their medical training and whether increased transparency in admissions processes may mitigate the unintended consequences of strategic self‐presentation or burnout. These findings could be extended beyond medical school to explore similar themes during residency applications.

## 5 Conclusion

Ultimately, our findings highlight how perceptions of admissions success shape pre‐medical identity formation. Recognizing participants' perceptions of limited transparency and implicit expectations that may encourage conformity to a homogenized archetype of the ‘successful medical school applicant,’ potentially at the expense of authenticity, may help inform more supportive and equitable approaches to career development and medical school admissions processes that can be explored.

## Author Contributions

**L. Canthiya:** conceptualization, investigation, writing – original draft, methodology, validation, writing – review and editing, formal analysis, project administration. **M. Wang:** data curation, investigation. **A. Bouriayee:** data curation, investigation. **R. Qamar:** investigation. **A. Acai:** supervision, formal analysis, validation, methodology, writing – review and editing, investigation, conceptualization, project administration, resources, data curation. **Preti BTB:** conceptualization, investigation, methodology, validation, visualization, writing – review and editing, formal analysis, project administration, data curation, supervision, resources.

## Funding

The authors have nothing to report.

## Conflicts of Interest

The authors declare no conflicts of interest.

## Data Availability

The data that support the findings of this study are available from the corresponding author upon reasonable request.

## Semi‐Structured Interview Guide Used to Explore Pre‐Medical Students' Perceptions of Medical School Admission Success and Decision Making.

### 1

**Table:**

Prompt 1	Why have you decided to pursue a career in medicine?
Prompt 2	What do you believe are the most important factors for getting into medical school and being successful? What are the biggest challenges you feel you will face in applying to medical school? Why do you think this?
Prompt 3	What steps are you taking to get into medical school? How did you decide to take these steps?
Prompt 4	What sources do you get information about applying to medical school from?
Prompt 5	What do you perceive makes a good doctor? How did you form this perception? Are there steps you are taking to become a good doctor?
